# Hemispheric Regional Based Analysis of Diffusion Tensor Imaging and Diffusion Tensor Tractography in Patients with Temporal Lobe Epilepsy and Correlation with Patient outcomes

**DOI:** 10.1038/s41598-018-36818-x

**Published:** 2019-01-18

**Authors:** Mahdi Alizadeh, Lauren Kozlowski, Jennifer Muller, Neha Ashraf, Shiva Shahrampour, Feroze B. Mohamed, Chengyuan Wu, Ashwini Sharan

**Affiliations:** 1 Department of Neurosurgery, Thomas Jefferson University, Philadelphia, PA USA; 2 Jefferson Integrated Magnetic Resonance Imaging Center, Department of Radiology, Thomas Jefferson University, Philadelphia, PA USA; 3 Sidney Kimmel Medical College, Thomas Jefferson University, Philadelphia, PA USA

## Abstract

Imaging in the field of epilepsy surgery remains an essential tool in terms of its ability to identify regions where the seizure focus might present as a resectable area. However, in many instances, an obvious structural abnormality is not visualized. This has created the opportunity for new approaches and imaging innovation in the field of epilepsy, such as with Diffusion Tensor Imaging (DTI) and Diffusion Tensor Tractography (DTT). In this study, we aim to evaluate the use of DTI and DTT as a predictive model in the field of epilepsy, specifically Temporal Lobe Epilepsy (TLE), and correlate their clinical significance with respect to postsurgical outcomes. A hemispheric based analysis was used to compare the tract density, as well as DTI indices of the specific regions of interest from the pathologic hemisphere to the healthy hemisphere in TLE patients. A total of 22 patients with TLE (12 males, 10 females, 22–57 age range) underwent either a craniotomy, Anterior Temporal Lobectomy (ATL), or a less invasive method of Selective Laser Amygdalohippocampectomy (SLAH) and were imaged using 3.0 T Philips Achieva MR scanner. Of the participants, 12 underwent SLAH while 10 underwent ATL. The study was approved by the institutional review board of Thomas Jefferson University Hospital. Informed consent was obtained from all patients. All patients had a diagnosis of TLE according to standard clinical criteria. DTI images were acquired axially in the same anatomical location prescribed for the T1-weighted images. The raw data set consisting of diffusion volumes were first corrected for eddy current distortions and motion artifacts. Various DTI indices such as Fractional Anisotropy (FA), Mean Diffusivity (MD), Radial Diffusivity (RD) and Axial Diffusivity (AD) were estimated and co-registered to the brain parcellation map obtained by freesurfer. 16 consolidated cortical and subcortical regions were selected as regions of interest (ROIs) by a functional neurosurgeon and DTI values for each ROI were calculated and compared with the corresponding ROI in the opposite hemisphere. Also, track density imaging (TDI) of 68 white matter parcels were generated using fiber orientation distribution (FOD) based deterministic fiber tracking and compared with contralateral side of the brain in each epileptic group: left mesial temporal sclerosis (LMTS) and right MTS (RMTS)). In patients with LMTS, MD and RD values of the left hippocampus decreased significantly using two-tailed t-test (p = 0.03 and p = 0.01 respectively) compared to the right hippocampus. Also, RD showed a marginally significant decrease in left amygdala (p = 0.05). DTT analysis in LMTS shows a marginally significant decrease in the left white matter supramarginal parcel (p = 0.05). In patients with RMTS, FA showed a significant decrease in the ipsilateral mesial temporal lobe (p = 0.02), parahippocampal area (p = 0.03) and thalamus (p = 0.006). RD showed a marginally significant increase in the ipsilateral hippocampus (p = 0.05) and a significant increase in the ipsilateral parahippocampal area (p = 0.03). Also, tract density of the ipsilateral white matter inferior parietal parcel showed a marginally significant increase compared to the contralateral side (p = 0.05). With respect to postsurgical outcomes, we found an association between residual seizures and tract density in five white matter segments including ipsilateral lingual (p = 0.04), ipsilateral temporal pole (p = 0.007), ipsilateral pars opercularis (p = 0.03), ipsilateral inferior parietal (p = 0.04) and contralateral frontal pole (p = 0.04). These results may have the potential to be developed into imaging prognostic markers of postoperative outcomes and provide new insights for why some patients with TLE continue to experience postoperative seizures if pathological/clinical correlates are further confirmed.

## Introduction

Neurologic diseases, such as epilepsy, often cause long-term debilitation for patients leading to deficits in brain function and frequent seizures^1^. Although epilepsy may arise from a vast number of causes including genetic, immune, and infectious, temporal lobe epilepsy (TLE) is among one of the most common types of this disease. Patients with TLE experience chronic, recurrent focal seizures that are typically unprovoked; however, patients with TLE do not always have simple partial seizures, and may also experience complex partial seizures that spread to other regions of the brain^2^. TLE is typically managed medically at the onset of the disease with various antiepileptic medications, but for those patients with intractable seizures failing to respond to numerous antiepileptic agents, surgery such as anterior temporal lobectomy (ATL) or selective laser amygdalohippocampectomy (SLAH) may be considered^2^. As previously mentioned, there are numerous causes that may lead to TLE, but the most frequent disease etiology that is refractory to medical management and responsive to surgical management is mesial temporal sclerosis (MTS), also commonly known as hippocampal sclerosis (HS)^3^.

Imaging in the field of epilepsy has been a lasting challenge, specifically, with regards to identifying the seizure focus as a resectable area for potential procedures^4^. This has created the opportunity for new approaches and imaging innovation in the field of epilepsy such as diffusion tensor imaging (DTI). DTI measures the magnitude and direction of the diffusion of water along 3 principle eigenvectors (x, y and z directions), and also provides insight into the microstructure of both gray and white matter by measuring water diffusion^5^.

The information provided by DTI acquisition allows for the quantification of various diffusion parametric maps as well as the generation of 3-dimentional (3D) white matter fiber tractography^5^. Diffusion tensor tractography (DTT) is a computational procedure that reconstructs major fiber bundles in 3D space based on their anisotropy properties. It is a visual and quantitative tool for presenting white matter tracts from DTI data^6,7^. White matter studies using DTI usually rely on the comparison of scalar measures that quantify the diffusion within a voxel. The most common measures, which are based on the DTI model, are fractional anisotropy (FA), which describes the degree of anisotropy within a voxel and can be attributed to the orientation of the axon fiber, and the mean diffusivity (MD), which quantifies the magnitude of diffusion. Other relevant scalar measures include axial diffusivity (AD) and radial diffusivity (RD) which estimate the diffusion parallel and perpendicular to the principle diffusion axis^6,7^. Reduction in FA represents the disorganization of axon fiber, leading to unrestricted or isotropic diffusion. Increased MD indicates a disorganization in structure, RD is a good indicator of myelin damage, and AD is an indicator of axonal damage such as axonal swelling, Wallerian degeneration, and axonal injury^8^.

DTI has grown to be a frequently utilized technique to study tractography in numerous neurological diseases and to visualize white matter pathways in the human brain due to its non-invasive nature. For instance, DTI has presented itself as an invaluable tool in measuring deficits in white matter such as in aging, and has been widely used as a clinical application in the study of Parkinson’s disease as well^9^.

There has also been a vast expansion in the use of DTI in the study of TLE, specifically enhancing our knowledge of epilepsy as a network disorder. The use of DTI for TLE patients has demonstrated disruptions in the structural integrity of cerebral white matter extending beyond just the temporal lobe and into several surrounding regions bilaterally, even in patients with unilateral MTS^10^. This has provided great insight for surgical planning in order to clearly lateralize the epileptic focus and aid in the delineation of disrupted white matter tracts extending into adjacent structures. However, more recent DTI studies describe the influence of these abnormalities in white matter tracts on cognitive networks leading to functional decline, specifically regarding language and memory^11^. The emerging DTI literature has also shown an understanding of the structural plasticity within the white matter tracts, demonstrating the utility of DTI in following adaptive changes and reorganization of function both pre and post-surgery. Although there has been development in DTI studies for TLE, a lack of clarity remains involving the correlation between the white matter abnormalities shown on tract density and the post-surgical outcomes with regards to seizure freedom.

Thus, there has been increasing application of DTI-based tractography as a clinical technique to better study pathological alterations to white matter structures and trace the axonal pathways involved in TLE. However, the available data regarding the relationship between preoperative DTI tractography and postoperative seizure outcome is scarce. In this study, we aim to evaluate the use of DTI as a predictive model for TLE in the setting of MTS. Note that up until now, there are still no reference value ranges for DTI and DTT measures in normal, healthy people. Therefore, related studies have been performed with the comparison between the contralateral (normal) and ipsilateral (abnormal) sides, or between the normal subjects (with no neurological diseases) and the patients^6^. A hemispheric based analysis was used to compare the DTI and DTT measures of specific regions of interest from the pathologic side to the healthy side in TLE patients. By correlating these quantitative measures with surgical outcomes through postoperative follow up, measuring the amount of seizure free time since the procedure, we propose the use of DTI and DTT as an imaging technique to generate a postoperative prognosis in TLE patients.

## Methods

### Participants

In this retrospective single-center study, a total of 22 patients with TLE (12 males, 10 females, 22–57 age range) underwent either anterior temporal lobectomy (ATL) or selective laser amygdalohippocampectomy (SLAH) and were scanned prior to surgery. There were no differences between patients undergoing ATL versus SLAH. The mean duration of drug resistant seizures was 18.3 years. The mean time of follow up after surgery was 1.3 years. On pre-operative MRI, 15 TLE patients were found to have left mesial temporal sclerosis (MTS) and 7 had right MTS. Of the participants, 12 underwent SLAH while 10 underwent ATL. At a 6 month follow up after surgical treatment, no seizures were reported from 12 patients, but 10 patients continued to experience seizures. The study was approved by the institutional review board (IRB) of Thomas Jefferson University Hospital. All methods were performed in accordance with the relevant guidelines and regulations approved by IRB. Informed consent was obtained from all patients. All patients had a diagnosis of TLE according to standard clinical criteria.

### Imaging

Subjects were scanned in a 3.0 T Philips Achieva MR scanner using an 8-channel head coil. DTI images were acquired axially using a single-shot echo planar imaging (EPI) sequence in the same anatomical location prescribed for T1-weighted images. The T1-weighted imaging parameters used were: FOV = 24.0 cm, voxel size = 1.0 × 1.0 × 1.0 mm^3^, matrix size = 512 × 512, TR = 12 ms, TE = 6 ms and slice thickness = 1 mm. The DTI parameters used were: FOV = 23.0 cm, number of directions = 15, number of reference scans (b0) = 1, b = 850 s/mm^2^, voxel size = 1.8 × 1.8 × 2.0 mm^3^, matrix size = 128 × 128, TR = 8.9 s, TE = 62 ms, number of averages = 1.

### DTI and T1 post-processing

**Figure 1:**
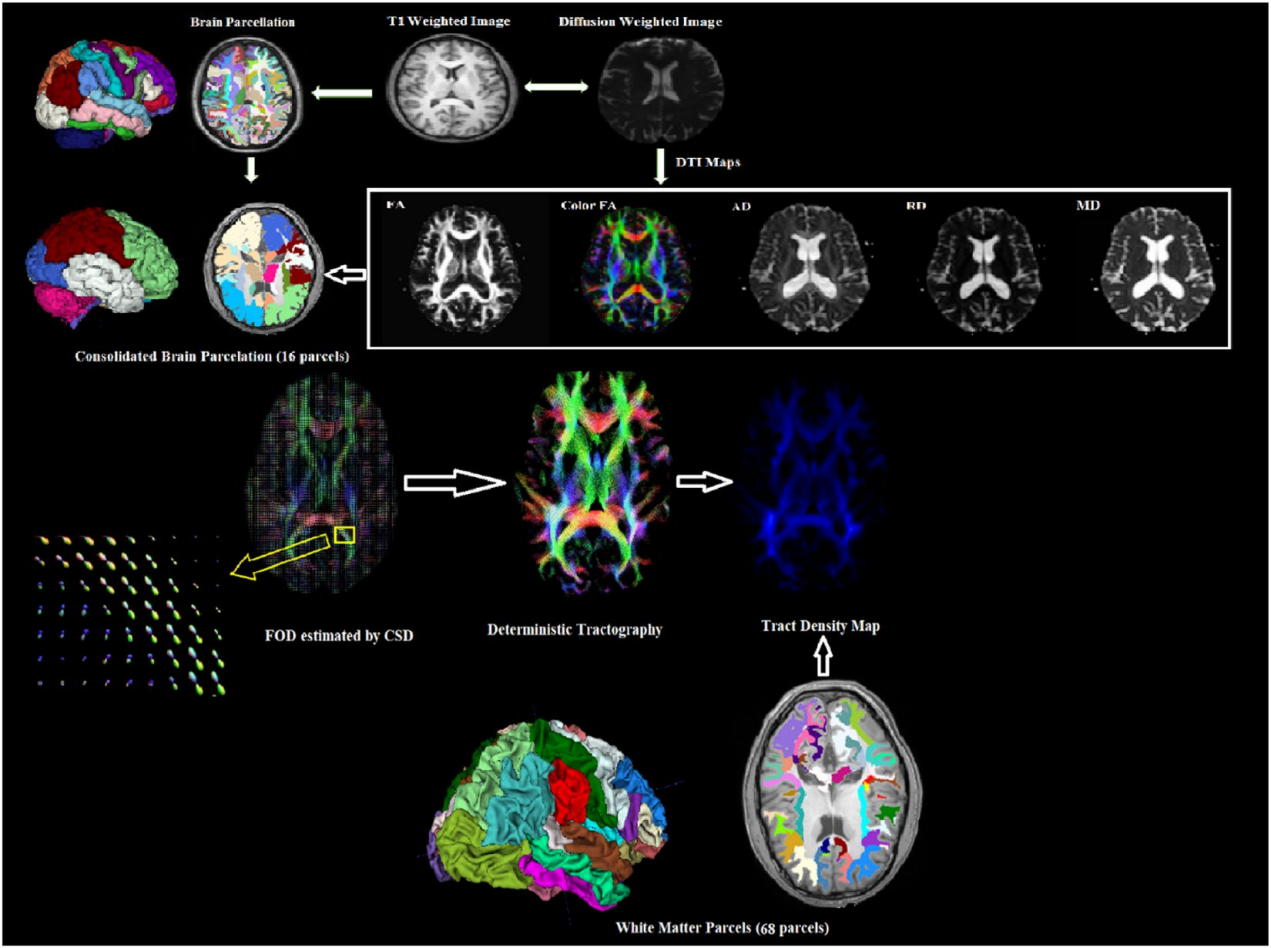
Illustration of the processing pipeline developed for DTI and DTT analysis.

The raw data set of diffusion volumes were first corrected for eddy current distortions using the FSL FDT diffusion toolbox and motion artifacts using FSL FLIRT (FMRIB’s Linear Image Registration Tool). Each directional diffusion image was aligned to the b0 volume (reference image) based on the 3D rigid body registration algorithm with 6 degrees of freedom and a correlation ratio as cost function to mitigate motion artifacts. Eigenvalues (λ_1_, λ_2_, λ_3_) and eigenvectors (*v*_1_, *v*_2_, *v*_3_) of the diffusion tensor matrix were computed from the pre-processed DTI volumes for each subject using FSL FDT diffusion toolbox. Various DTI indices such as FA, MD, RD and AD were generated. The FA, MD, RD and AD maps for TLE patients were then co-registered to the brain parcellation map in freesurfer space based on the affine transformation algorithm and normalized mutual information as cost function implemented in SPM12 (Fig. 1).

FreeSurfer (http://surfer.nmr.mgh.harvard.edu) was used to obtain surface meshes of the boundary between grey matter and white matter from T1 anatomical brain images. FreeSurfer provides parcellation of anatomical regions of the cortices and subcortical regions in both hemispheres. 16 consolidated cortical and subcortical regions were selected as regions of interest (ROIs) by a functional neurosurgeon. These consolidated regions included cingulate, hippocampus, amygdala, parahippocampal, basal ganglia, mesial temporal, lateral temporal, thalamus, cerebellum, precuneus, entorhinal, cuneus, insula, frontal lobe, parietal lobe and occipital lobe.

DTI values for each ROI were calculated and compared with the corresponding ROIs in the opposite hemisphere. Additionally, track density imaging (TDI) of 68 white matter parcels were generated using fiber orientation distribution (FOD) based deterministic fiber tracking and compared with the contralateral side of the brain in each epileptic group (LMTS and RMTS). FOD was estimated using the constrained spherical deconvolution (CSD) model implemented in MRtrix (http://www.mrtrix.org/), which has been shown to provide robust results compared to other existing deterministic tractography algorithms^12–14^.

## Results

### DTI and tractography findings

The mean values of pre-operative FA, MD, AD, RD and tract density for selected ROIs were calculated and compared to the contralateral side of the brain. In patients with LMTS (Table 1), MD and RD values of the left hippocampus were found to be significantly lower when compared to the right hippocampus using a two-tailed t-test (p = 0.03 and p = 0.01 respectively). Additionally, RD showed a marginally significant decreased in left amygdala (p = 0.05) compared to the right amygdala in patients with LMTS. DTT analysis in LMTS patients showed a marginally significant decrease in the left white matter supramarginal parcel (p = 0.05). In patients with RMTS (Table 2), FA was significantly lower in the ipsilateral mesial temporal lobe (p = 0.02), parahippocampal region (p = 0.03) and thalamus (p = 0.006). RD showed a marginally significant increase in the ipsilateral hippocampus (p = 0.05) and significant increase in the ipsilateral parahippocampal region (p = 0.03). Additionally, the tract density of the ipsilateral white matter inferiorparietal parcel demonstrated a marginally significant increase when compared to the contralateral side (p = 0.05).

**Table 1:** DTI and DTT findings in patients with LMTS.

		FA	MD (10^−3^mm^2^/s)	AD (10^−3^mm^2^/s)	RD (10^−3^mm^2^/s)	Tract Density
Hippocampus	LH	0.21 ± 0.005 (p = 0.87)	1.22 ± 2.51E-5 (p = 0.03)	1.45 ± 4.8E-5 (p = 0.07)	1.08 ± 1.5E-5 (p = 0.01)	—
	RH	0.21 ± 0.004	1.09 ± 1.28E-5	1.3 ± 2.59E-5	0.97 ± 5.8E-6	—
Amygdala	LH	0.21 ± 0.003 (p = 0.66)	1.02 ± 3.89E-5 (p = 0.18)	1.23 ± 4.66E-5 (p = 0.2)	0.89 ± 1.8E-5 (p = 0.05)	—
	RH	0.22 ± 0.004	0.9 ± 4.22E-5	1.1 ± 4.4E-5	0.8 ± 1.2E-5	—
WM supramarginal parcel	LH	—	—	—	—	148.22 ± 12.61 (p = 0.05)
	RH	—	—	—	—	240.64 ± 15.82

LH = Left Hemisphere; RH = Right Hemisphere.

**Table 2:** DTI and DTT findings in patients with RMTS.

		FA	MD (10^−3^mm^2^/s)	AD (10^−3^mm^2^/s)	RD (10^−3^mm^2^/s)	Tract Density
Hippocampus	LH	0.2 ± 6.06E-4 (p = 0.98)	1.05 ± 4.5E-6 (p = 0.09)	1.26 ± 1.24E-5 (p = 0.2)	0.95 ± 2.17E-6 (p = 0.05)	—
	RH	0.21 ± 1.2E-3	1.12 ± 2.6E-6	1.34 ± 7.94E-6	1.01 ± 1.38E-5	—
Mesial temporal lobe	LH	0.27 ± 2.37E-4 (p = 0.02)	0.95 ± 4.2E-6 (p = 0.09)	1.2 ± 7.89E-6 (p = 0.19)	0.82 ± 2.7E-6 (p = 0.06)	—
	RH	0.23 ± 5.27E-4	1.03 ± 8E-6	1.28 ± 1.11E-5	0.91 ± 7.29E-6	—
Para hippocampal	LH	0.27 ± 2.4E-3 (p = 0.03)	0.9 ± 1.5E-5 (p = 0.11)	1.16 ± 3.46E-5 (p = 0.34)	0.7 ± 7.73E-6 (p = 0.03)	—
	RH	0.21 ± 4.6E-4	1.03 ± 1.5E-5	1.26 ± 3.4E-5	0.9 ± 9.93E-6	—
Thalamus	LH	0.36 ± 5.28E-4 (p = 0.006)	0.88 ± 4.4E-6 (p = 0.19)	1.22 ± 4.71E-6 (p = 0.55)	0.71 ± 4.33E-6 (p = 0.08)	—
	RH	0.32 ± 5.06E-5	0.94 ± 6.9E-6	1.25 ± 7.32E-6	0.8 ± 6.52E-6	—
WM inferiorparietal parcel	LH	—	—	—	—	160.33 ± 76.72 (p = 0.05)
	RH	—	—	—	—	271.86 ± 84.44

LH = Left Hemisphere; RH = Right Hemisphere.

### DTI and tractography relation to clinical outcomes

**Figure 2:**
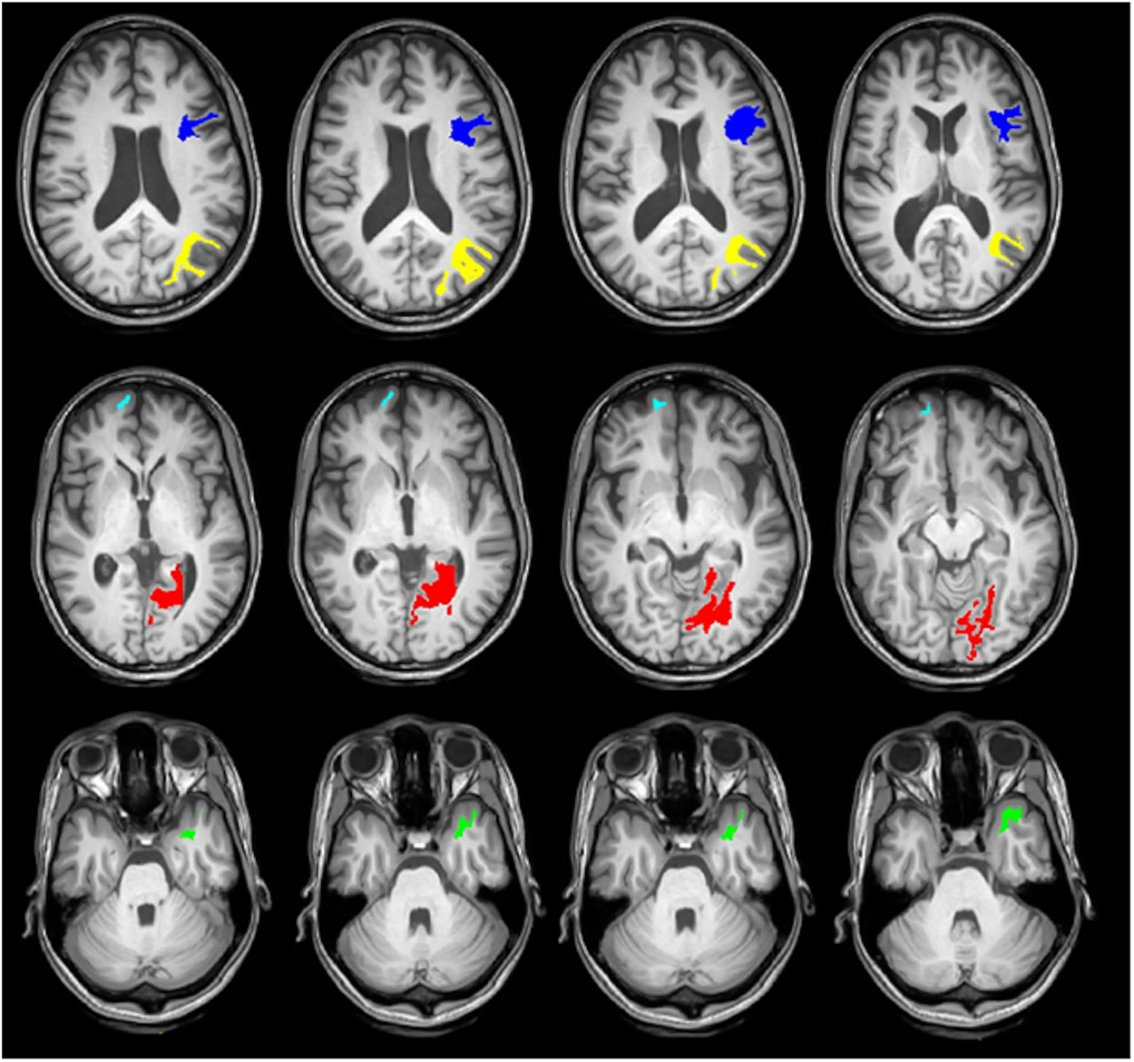
Cross-sectional images of the white matter parcels with significant differences in tract density (p < 0.05) between responders (n = 12) and non-responders (n = 10) to the surgical treatments. These regions include ipsilateral lingual (red), ipsilateral temporal pole (green), ipsilateral pars opercularis (blue), ipsilateral inferior parietal (yellow) and contralateral frontal pole (cyan).

When comparing the DTI indices between TLE responders (n = 12) and non-responders (n = 10) to surgical treatments, no significant differences were observed in any of the consolidated parcels. However, looking at the tract density of white matter parcels, significant increases were observed in five distinct white matter parcels (Table 3) in non-responders. The anatomical locations of these parcels are shown in Fig. 2. These regions include ipsilateral lingual (p = 0.04), ipsilateral temporal pole (p = 0.007), ipsilateral pars opercularis (p = 0.03), ipsilateral inferior parietal (p = 0.04) and contralateral frontal pole (p = 0.04).

**Table 3:** Tract density measures of WM parcels with significant differences between two groups.

	Non- Responders	Responders	Prob > |t|
Ipsilateral lingual	255.36 ± 84.55	175.78 ± 63.57	0.04
Ipsilateral temporal pole	426.52 ± 119.8	279.86 ± 50.75	0.007
Ipsilateral pars opercularis	257.73 ± 47.84	192.87 ± 58.38	0.03
Ipsilateral inferior parietal	309.55 ± 103.73	198.56 ± 76.21	0.04
contralateral frontal pole	282.74 ± 73.92	175.42 ± 103.45	0.04

## Discussion

This study shows that DTI and DTT analysis has the potential to aid in the delineation of disrupted white matter tracts extending into adjacent structures. However, DTI studies describe the influence of these abnormalities in white matter tracts within cognitive networks leading to functional decline, specifically regarding language and memory^2^. The emerging DTI literature has also shown an understanding of the structural plasticity within the white matter tracts, demonstrating the utility of DTI in following adaptive changes and reorganization of function both pre and post-surgery. This study helps to better understand the pathological alterations in white matter structures, and in tracing axonal pathways involved in TLE. However, available data regarding the relationship between preoperative DTI tractography and postoperative seizure outcome remains scarce.

Left and right MTS patients exhibit similar functional and clinical dysfunction, such as depressed mood, emotional dysregulation, and memory deficits^15^. However, previous studies indicated that the left and right MTS patients involved distinct pathological and etiological substrates^15^. Anatomical connectivity analysis showed different connectivity patterns in cortical-limbic networks as well as within the cerebellum in left MTS compared to right MTS^16^. Tractography analysis also suggests bilateral, widespread white matter changes in patients with left TLE, and mostly unilateral white matter changes in patients with right TLE compared to controls^17^.

Patients with LMTS had significantly lower MD and RD values of the left hippocampus when compared to the contralateral side, likely due to the sclerosis of this structure in the mesial temporal lobe of the pathological hemisphere^18^. Additionally, as the hippocampus is a part of the limbic system, it plays a crucial role in long-term memory and spatial memory allowing for navigation. This can lead to clinical deficits in LMTS patients such as failure to consolidate short into long term memories caused by the hippocampal sclerosis.

In addition to the hippocampal findings, the RD value in patients with LMTS was significantly lower in the left amygdala compared to the right amygdala. This significance is suspected to be due to the amygdala’s involvement in the limbic system along with the hippocampus^19^. Although MTS typically involves sclerosis of the hippocampus, it is not unreasonable to conclude that this pathological process will involve other structures located medially within the temporal lobes of the brain as well as neuronal connections projecting to other structures involving the limbic system, such as the amygdala. Like the hippocampus, the amygdala plays a primary role in memory consolidation, both memory formation and storage, but also processes emotional reactions, which similarly, leads to clinical deficits in LMTS patients^20^. As a part of the limbic system the amygdala also sends projections to numerous other structures within the brain such as the hypothalamus and thalamic nuclei which may be involved in the clinical pathology as well.

In addition to the DTI findings in LMTS patients, DTT analysis yielded a marginally significant difference showing a decrease in the left white matter supramarginal parcel compared to the right. The supramarginal gyrus is not part of the limbic system like the other structures showing significant decreases in DTI values. However, the supramarginal gyrus is located in the parietal lobe which is adjacent to the temporal lobe^21^. With extension of the sclerotic structure beyond the temporal lobe in the pathologic hemisphere into surrounding areas, the supramarginal gyrus may be affected, as in this case, Additionally, as neuronal connections traverse the brain to reach the mesial temporal lobe, fibers may cross through or near the supramarginal gyrus as well leading to the significant decrease in the DTT versus the DTI value on the diseased side.

Patients with RMTS had a significantly lower FA value in the ipsilateral mesial temporal lobe, parahippocampal area, and thalamus, with ipsilateral referring to the pathological hemisphere. The RD value was significantly higher in the ipsilateral hippocampus and parahippocampal area as well. As previously described, the lower values in the mesial temporal lobe are likely due to the presence of mesial temporal sclerosis in the pathological hemisphere^18^. The presence of mesial temporal sclerosis also explains the lower values seen in the parahippocampus as well as it is the region of the brain surrounding the hippocampus, also involved in the limbic system, and has been shown to be affected by this sclerotic process in temporal lobe epilepsy^22^. The increase in ipsilateral RD values for the hippocampus and parahippocampus is related to the meaning of the RD value as it measures increases in white matter with demyelination which occurs with MTS. Lower FA values in DTI analysis were also exhibited in the thalamus, likely due to the numerous neuronal connections between the thalamus and hippocampus as the thalamus acts as a relay station for information between different areas within the brain^23^. This relationship between the thalamus and hippocampus may have led to this result due to extension of the hippocampal sclerosis beyond just the hippocampus and involving the surrounding structures and connections.

Additionally, the ipsilateral inferior parietal parcel was marginally significant in DTT analysis when compared to the contralateral side in RMTS patients. This finding may be consistent with the fact that the inferior parietal lobule is adjacent to the temporal lobe, and the angular gyrus, a part of the inferior parietal lobule, is a direct extension of the middle temporal gyrus^18^. Therefore, due to the close proximity and likely axonal connections between the inferior parietal parcel and the mesial temporal lobe, it is likely that this area of the brain was affected by an extension of MTS, especially involving the fibers crossing between structures in DTT analysis.

In relation to clinical outcomes, no significant difference was seen in any consolidated parcels in comparison to TLE patients who responded to surgical treatments versus those who did not respond. However, significant increases are observed in surgical treatment non-responders when looking at tract density of the following white matter parcels: ipsilateral lingual, ipsilateral temporal pole, ipsilateral pars opercularis, ipsilateral inferior parietal, and contralateral frontal pole. The significant decreases exhibited in tractography on the ipsilateral hemisphere as the pathological process are likely attributed to direct effects of the sclerotic disease such as the ipsilateral temporal pole in TLE patients^18^. These findings may also be attributed to extensions of the disease process/disruptions in connections to adjacent structures, for instance, the ipsilateral lingual gyrus which joins the parahippocampal gyrus and is a continuation of the tentorial surface of the temporal lobe^22^.

While this yielded several novel results, there are limitations to the study that may lead to additional findings in the future. For instance, all images used in this study were pre-operative scans. In the future, analyzing the post-operative scans and correlating the results with clinical outcomes as well as surgical changes may also result in novel findings. Correlating both pre and post-operative scans with cognitive measures, such as language and memory, may also lead to additional novel findings. Thus, the significant changes observed may be skewed due to different factors such as age, gender, duration of drug resistant seizures, duration of seizure onset, follow up delays and so on. By having a larger sample size, the analysis can be done as a function of these covariates to minimize DTI and DTT inter-subject variability. Although this was not the case, each analysis showed clear differences and patterns, supporting the conclusion that DTI and DTT have the potential to be used as prognostic markers and differentiate the epileptogenic networks between responders and non-responders. Also, use of antiepileptic drugs and heterogeneity in TLE etiology have been shown to affect DTI. Earlier age of onset has been associated with decreased hippocampal volumes. As a population-based study of TLE, patients with several different pathologies were excluded in this study. However, heterogeneity of pathology, disease duration, seizure frequency and severity, and age of disease onset may also lead to attenuated detection of changes in TLE. Further studies on more homogeneous groups are needed to control for these potential confounders^24,25^.

There is also the need to acquire high resolution DTI at a higher field strength, with improved radiofrequency coils and multiband DTI techniques, which will allow for the imaging of small voxels while still maintaining a relatively short imaging time. Using a voxel size of 1.8 × 1.8 × 2 mm^3^, it is certainly possible that a single voxel will contain numerous fibers which may not have the same orientations. Different tracts may cross within a single voxel; therefore, imprecise data may be obtained.

The fiber tracts of the brain were generated using a deterministic streamline approach. This method has proven to be an effective algorithm capable of estimating the trajectories of white matter tracts in the brain and the cord^26–28^. However, deterministic fiber tracking has limitations, particularly in voxels where fibers are crossing, bending or kissing. The origin of this crossing fibers challenge lies in the fact that DTI requires a relatively low number of diffusion weighted directions, causing the tract to terminate or be generated inaccurately. One way to resolve this problem is to use advanced diffusion imaging techniques with higher number of gradient directions such as High Angular Resolution Diffusion Imaging (HARDI) or Neurite Orientation Dispersion and Density Imaging (NODDI)^29,30^ in conjunction with probabilistic fiber tracking. Probabilistic fiber tracking evaluates all possible propagation directions to generate the neural tracts. With more gradient directions and therefore a higher angular resolution, more accurate production of fibers is possible. The optimal number of diffusion weighted directions is still unresolved and depends on different factors such as the robustness of the post processing algorithms, voxel size, reasonable acquisition time for clinical applications and scan parameters (e.g., TE)^12,13^.

## Conclusion

These results may have the potential to be developed into imaging prognostic markers of postoperative outcomes and provide new insights for why some patients with TLE continue to experience postoperative seizures if pathological/clinical correlates are further confirmed. On the contrary, areas predicting unfavorable postsurgical outcome were distinct, suggesting different configuration of epileptogenic networks between responders and non-responders. These preliminary results are very encouraging and warrant further studies with a larger population.
